# Time trends in surgical provision and cancer-specific outcomes in patients with stage T2-3 kidney cancer: a SEER-based study

**DOI:** 10.3389/fsurg.2024.1370702

**Published:** 2024-04-29

**Authors:** Zhuo Song, Jizhang Xing, Zhijia Sun, Xiaoli Kang, Hongzhao Li, Gang Ren, Yingjie Wang

**Affiliations:** ^1^ Department of Radiotherapy, Air Force Medical Center, Air Force Medical University, Beijing, China; ^2^ Department of Urology, Air Force Medical Center, Air Force Medical University, Beijing, China; ^3^ Department of Urology, The General Hospital of the People’s Liberation Army, Beijing, China; ^4^ Department of Radiotherapy, Peking University Shougang Hospital, Beijing, China

**Keywords:** kidney cancer, nephrectomy, cancer-specific outcomes, trends, SEER

## Abstract

**Background and objective:**

Surgery is the primary therapy that crucially affects the survival of patients with kidney cancer (KC). However, pertinent surgical decision criteria for individuals with stage T2-3 KC are lacking. This study aimed to display the practical choices and evolving trends of surgical procedures and elucidate their implied value.

**Methods:**

Through the Surveillance, Epidemiology, and End Results (SEER) dataset, the levels and evolving trends of different surgical methods were examined to determine cancer-specific risk of death (CSRD). Additionally, stratification analysis and survival rate analysis were performed to explore the effectiveness of partial nephrectomy (PN).

**Results:**

In this study, 9.27% of patients opted for PN. Interestingly, an upward trend was observed in its decision, with an average annual percentage change (AAPC) of 7.0 (95% CI: 4.8–9.3, *P* < 0.05). Patients who underwent PN and were in a relatively less severe condition exhibited more favorable CSRD levels (0.17–0.36 vs. 0.50–0.67) and an improvement trend compared with those who underwent radical nephrectomy (RN) (AAPC: −1.9 vs. −0.8). Further analysis showed that the levels of CSRD and survival rates for patients opting for different surgical methods followed a similar pattern.

**Conclusions:**

This study showed that RN was still the most common surgical method. Patients with stage T2-3 KC had an increasing preference for PN and exhibited more favorable cancer-related survival outcomes, which underscores the need for further investigation and validation.

## 1 Introduction

Kidney cancer (KC) is a malignancy with relatively favorable prognostic outcomes, accounting for 2.2% of all cancer cases and 1.8% of all cancer-specific deaths (CSDs) globally (1). The incidence of KC steadily increases with age and grows even worse with continuous aging (2). Radical nephrectomy (RN) is the predominant treatment modality for KC. The advancement of this therapy has contributed greatly to the decrease in KC-related mortality over the recent decades (3, 4). Nevertheless, owing to the relatively positive prognosis and earlier detection of smaller tumors, trends in the surgical modalities for KC are evolving. These trends are increasingly focusing on preserving the organ and minimally invasive techniques, such as partial nephrectomy (PN) assisted with a robot or laparoscope (3, 5).

With the superiority of preserving organ function to RN, the effectiveness exploration and technical optimization preference for PN have been hot topics in the field of surgery methods for KC in the past two decades. Compared with RN, PN is known for its ability to preserve kidney function, ultimately translating into survival benefits. Therefore, it has become the preferred and standard treatment for patients with stage T1a KC (6, 7). However, the current evidence supporting the superiority of PN to RN is primarily based on retrospective cohort studies. For T2-3 patients with relatively large tumors, there is even limited evidence concerning their advantages from prospective randomized controlled trials (8, 9). PN also presents its own set of challenges, including increased surgical complexity and a higher likelihood of positive margins. This may lead to a less favorable prognosis, particularly for patients with pre-existing health conditions (5). Studies in North America have reported that the effect of PN vs. RN on patients of KCs with large tumors may be limited and that tumor outcomes may be more closely associated with the nature of the disease (10, 11). At present, some studies have elucidated the advantages of PN and RN from different perspectives and explored different aspects, including the specific population such as elderly patients (12–14), factors from kidney function levels or others (15, 16), and the transition from planned PN to RN (17). The optimal surgical decision for patients with stage T2-3 KC and the suitable patient population have not been comprehensively assessed and discussed in clinical settings.

Therefore, this study aimed to analyze how the choice of surgical methods for stage T2-3 KC affects their cancer-specific outcomes and their variation trends, containing cancer-specific risk of death (CSRD) and survival rates at various time points, aiming to provide possible novel insights into the surgical selection among such patients.

## 2 Materials and methods

### 2.1 Study population and data sources

The analysis data were obtained from the Surveillance, Epidemiology, and End Results (SEER) database. Research cases were identified from the case list in “Incidence-SEER Research Plus Data, 18 Registries, Nov 2020 Sub (2000–2018)”, based on “site and morphology CS Schema AJCC 6th Edition” = “Kidney”. The patient inclusion criteria were as follows: individuals with a confirmed pathological diagnosis, T staging of T2-3 (patients diagnosed before 2015 were classified according to AJCC 6th, thereafter by EOD 2018), local or regional KC, and a clearly defined surgical approach. The surgical approaches were divided into five categories: no surgery [code: 00], local tumor destruction with ablation [code: 11–15], local tumor excision with ablation [code: 21–25], PN [code: 30], and RN [code: 40–80]. The variables were collected according to: (i) age at diagnosis, (ii) sex (female/male), (iii) age (<65 years/≥65 years), (iv) marital status (single/married/unknown), (v) histological type (clear cell adenocarcinoma/papillary adenocarcinoma/others/renal cell carcinoma but type unknown, (vi) grade (I/II/III/IV/unknown), (vii) laterality (right/left/other), (viii) stage T (T2/T3), (ix) N positive (no/yes/unknown), (x) size of tumor, (xi) surgical information, (xii) radiotherapy (beam radiation, no/yes/other types), (xiii) chemotherapy (no/yes/unknown), (xiv) survival months, (xv) survival status (alive/death), and (xvi) specific death status (no/yes/unknown). This study was conducted in adherence with the Data Use Agreement from the National Cancer Institute and considered exempted research by our institution.

### 2.2 Cancer-related outcomes used in this study

Two concepts related to cancer-specific outcomes were considered: CSD rate, defined as the ratio of the number of individuals who died from a specific cancer to the total population of that surgical type, and CSRD, defined as the ratio of the CSD rate to the ratio of deaths within that population. This approach considers the changes in CSRD over different years because of the potential differences in the proportion of deaths. Considering that the efficacy of some cancer treatments may predominantly involve postponing death, survival rates at various time points were also used to gain additional insights into treatment effectiveness.

### 2.3 Statistical analysis

Firstly, patients were divided into different subgroups based on their diagnosis year and surgical methods they selected. Descriptive statistical methods were used to determine the levels and trends of how patients with stage T2-3 KC selected various surgical methods and their corresponding CSRD over the years. The joinpoint regression method was used to calculate annual percentage change (APC) and average annual percentage change (AAPC) for determining the variation trends in the proportion of surgery selection and CSRDs. Chi-squared test or Wilcoxon rank-sum test was used to compare disease severity across various surgical groups in Table 1. Logistic regression analysis and stratified analysis were performed to identify the risk factors for CSD and assess how these factors affect CSRDs. The occurrence of CSD was considered a dependent variable, whereas the surgical method was an independent variable. Lastly, the levels and trends of survival rates at various time points were analyzed for three surgical modalities utilizing the survival package of R software (version 4.2.1 for Windows). Statistical significance was set at *P* < 0.05.

**Table 1:** Demographic characteristics of the five surgical groups.

	Total	NN	DA	EA	PN	RN	*P*
*N* (%)	32,135	657 (2.0)	41 (0.1)	23 (0.1)	2,980 (9.3)	28,434 (88.5)	
Age ≥65 (%)	14,758 (45.9)	450 (68.5)	29 (70.7)	14 (60.9)	1,365 (45.8)	12,900 (45.4)	<0.001
Sex = Female (%)	10,489 (32.6)	229 (34.9)	17 (41.5)	8 (34.8)	868 (29.1)	9,367 (32.9)	<0.001
Marital status (%)							
Single	10,602 (33.0)	306 (46.6)	15 (36.6)	8 (34.8)	885 (29.7)	9,388 (33.0)	<0.001
Married	20,144 (62.7)	314 (47.8)	23 (56.1)	14 (60.9)	1,945 (65.3)	17,848 (62.8)	
Unknown	1,389 (4.3)	37 (5.6)	3 (7.3)	1 (4.3)	150 (5.0)	1,198 (4.2)	
Histological type							
Clear cell adenocarcinoma	19,647 (61.1)	274 (41.7)	19 (46.3)	11 (47.8)	1,544 (51.8)	17,799 (62.6)	<0.001
Papillary adenocarcinoma	3,223 (10.0)	54 (8.2)	4 (9.8)	3 (13.0)	617 (20.7)	2,545 (9.0)	
Others	3,968 (12.3)	103 (15.7)	3 (7.3)	2 (8.7)	405 (13.6)	3,455 (12.2)	
RCC but type unknown	5,297 (16.5)	226 (34.4)	15 (36.6)	7 (30.4)	414 (13.9)	4,635 (16.3)	
Grade							
I	1,538 (6.1)	47 (17.9)	3 (13.0)	2 (15.4)	183 (8.1)	1,303 (5.7)	<0.001
II	10,570 (41.7)	104 (39.7)	15 (65.2)	8 (61.5)	1,071 (47.7)	9,372 (41.1)	
III	9,987 (39.4)	87 (33.2)	4 (17.4)	2 (15.4)	841 (37.4)	9,053 (39.7)	
IV	3,227 (12.7)	24 (9.2)	1 (4.3)	1 (7.7)	152 (6.8)	3,049 (13.4)	
Stage T = T3 (%)	19,871 (61.8)	329 (50.1)	31 (75.6)	18 (78.3)	2,162 (72.6)	17,331 (61.0)	<0.001
*N* positive							
No	29,698 (92.4)	446 (67.9)	40 (97.6)	23 (100)	2,884 (96.8)	26,305 (92.5)	<0.001
Yes	1,731 (5.4)	163 (24.8)	1 (2.4)	0 (0)	38 (1.3)	1,529 (5.4)	
Unknown	706 (2.2)	48 (7.3)	0 (00	0 (0)	58 (1.9)	600 (2.1)	
Radiotherapy (Beam radiation)							
No	31,787 (98.9)	632 (96.2)	40 (97.6)	23 (100)	2,979 (100.0)	28,113 (98.9)	<0.001
Yes	323 (1.0)	22 (3.3)	0 (0)	0 (0)	1 (0.0)	300 (1.1)	
Other types	25 (0.1)	3 (0.5)	1 (2.4)	0 (0)	0 (0.0)	21 (0.1)	
Chemotherapy = Yes (%)	1,587 (4.9)	149 (22.7)	2 (4.9)	0 (0)	62 (2.1)	1,374 (4.8)	<0.001
Specific death (%)							
No	19,843 (61.7)	135 (20.5)	19 (46.3)	16 (69.6)	2,317 (77.8)	17,356 (61.0)	<0.001
Yes	7,354 (22.9)	350 (53.3)	10 (24.4)	4 (17.1)	281 (9.4)	6,709 (23.6)	
Other death	4,938 (15.4)	172 (26.2)	12 (29.3)	3 (13.0)	382 (12.8)	4,369 (15.4)	
Tumor size [median (IQR)]	80.0 [60.0, 100.0]	84.0 [73.0, 100.0]	40.0 [31.0, 82.0]	41.0 [28.5, 67.0]	47.0 [30.0, 80.0]	81.0 [65.0, 102.0]	<0.001

NN, no nephrectomy; DA, local tumor destruction with ablation; EA, local tumor excision with ablation; PN, partial nephrectomy; RN, radical nephrectomy; RCC, renal cell carcinoma.

## 3 Results

### 3.1 Proportion and trends in the population

**Figure 1:**
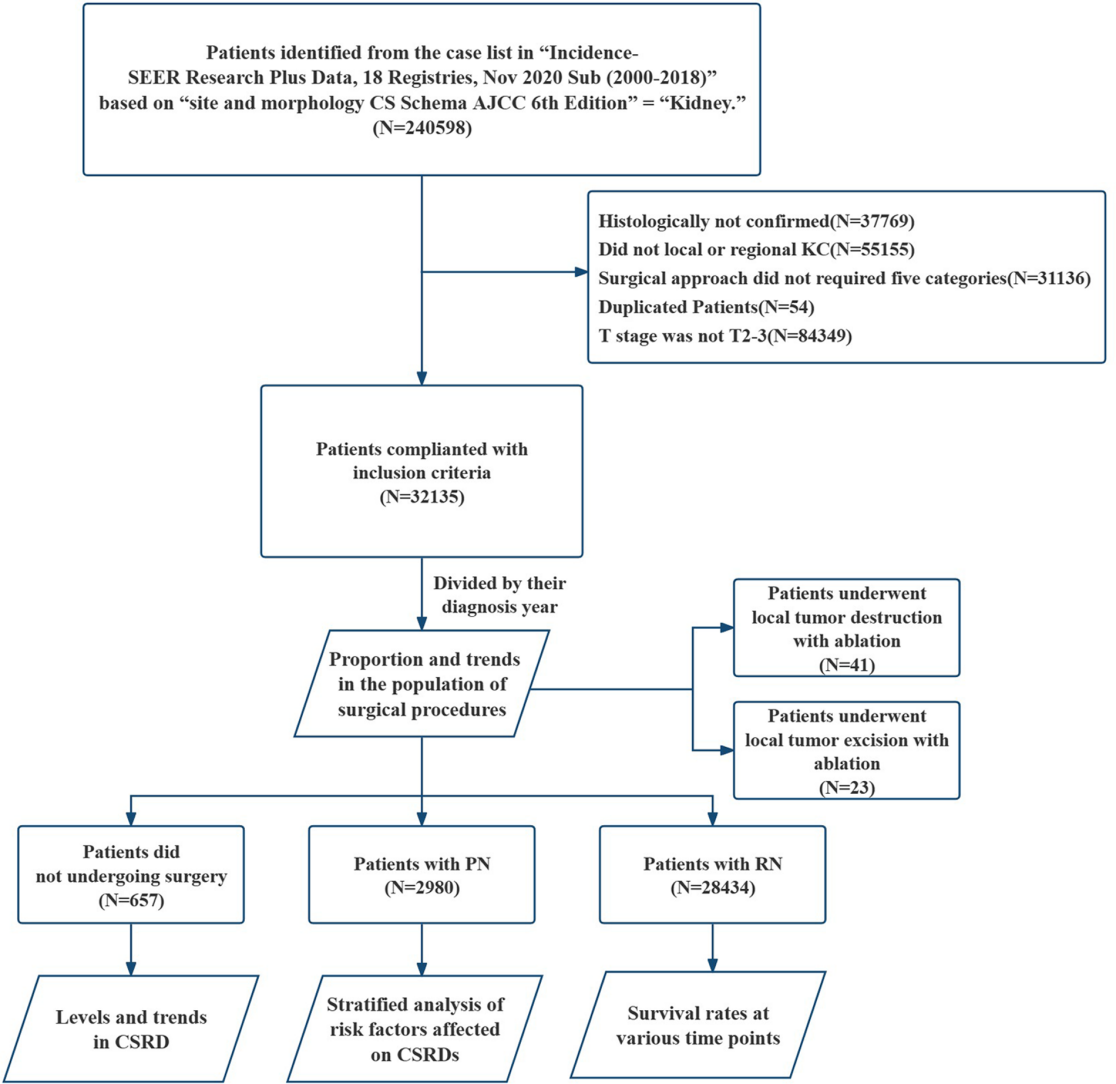
Flowchart on the patient selection from the SEER database. SEER, the surveillance, epidemiology, and end results dataset; KC, kidney cancer; PN, partial nephrectomy; RN, radical nephrectomy; CSRD, cancer-specific risk of death.

A total of 32,135 patients were included in this study (Figure 1), and 45.9% of patients aged 65 or older (Table 1). Patients with stage T2 and T3 accounted for 38.16% and 61.84% of the total population, respectively, and the male–female ratio was approximately 2:1. The most common pathological types were clear cell adenocarcinoma and papillary adenocarcinoma, accounting for 61.14% and 10.02%, respectively. In addition, 16.49% of the cases were identified as renal cell carcinoma, but the type was unknown. The remaining 12.35% of patients developed primarily rare renal cell carcinoma. Very few cases of KC have been observed, but they are all non-renal cell carcinomas. There were 12,292 deaths and 7,354 CSDs, accounting for 38.25% and 22.88% of the total population, respectively. Additionally, 88.48% of patients opted for RN, whereas only 9.27% opted for PN and 2.04% did not opt for surgery. The proportion of the population that underwent local tumor ablation (local tumor destruction or excision with ablation) was only 0.20%.

**Figure 2:**
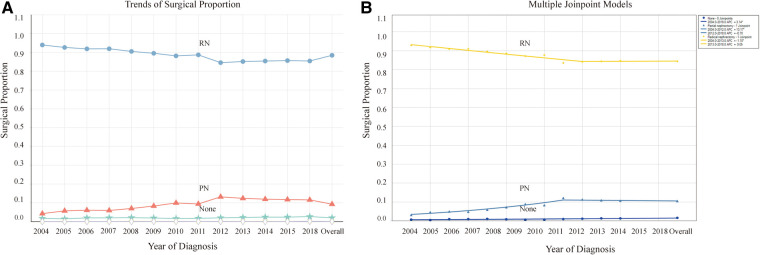
Proportion and trends in the population undergoing different surgical methods. (**A**) Proportion in the population undergoing different surgical methods and their variation trends over time. (**B**) Trends of the proportion undergoing different surgical methods using joinpoint regression analysis. *P < 0.05.

RN has been the predominant treatment modality for patients with stage T2-3 KC. However, it displayed a decreasing trend and then gradually reached stability. While PN has presented an opposite trend, it initially increased and then stabilized, with 2012 as the turning point. By 2018, PN accounted for approximately 11.6% of all cases, with an AAPC of 7.0 (95% CI: 4.8–9.3, *P* < 0.05) (Table 2, Figure 2B). These trends indicate an increasing emphasis on using PN as a surgical method for patients with stage T2-3 KC.

**Table 2:** Changing trends in surgical procedures and CSRD across different surgical methods from 2004 to 2018.

	Surgery methods	Year of diagnosis	APC [95% CI]	AAPC [95% CI]	*t*	*P*
Surgical selection	NN	2004–2018	3.14 [1.39, 4.93]*	3.1 [1.4, 4.9]*	3.97	0.00
	PN	2004–2012	13.17 [10.26, 16.17]*	7.0 [4.8, 9.3]*	10.94	0.00
		2012–2018	−0.70 [−5.09, 3.89]		−0.36	0.73
	RN	2004–2013	−1.10 [−1.40, −0.79]*	−0.7 [−1.0, −0.4]*	−8.31	0.00
		2013–2018	0.05 [−0.75, 0.86]		0.14	0.89
CSRD	NN	2004–2018	9.10 [7.09, 11.14]*	9.1 [7.1, 11.1]*	10.33	0.00
	PN	2004–2018	-1.92 [−5.20, 1.48]	-1.9 [−5.2, 1.5]	-1.25	0.24
	RN	2004–2014	2.03 [1.47, 2.59]*	-0.8 [−1.4, −0.1]*	8.46	0.00
		2014–2018	-7.42 [−9.57, −5.22]*		-7.57	0.00

NN, no nephrectomy; PN, partial nephrectomy; RN, radical nephrectomy; APC, annual percentage change; AAPC, average annual percentage change.

* *P *< 0.05.

### 3.2 Levels and trends in CSRD and demographic characteristics of patients

**Figure 3:**
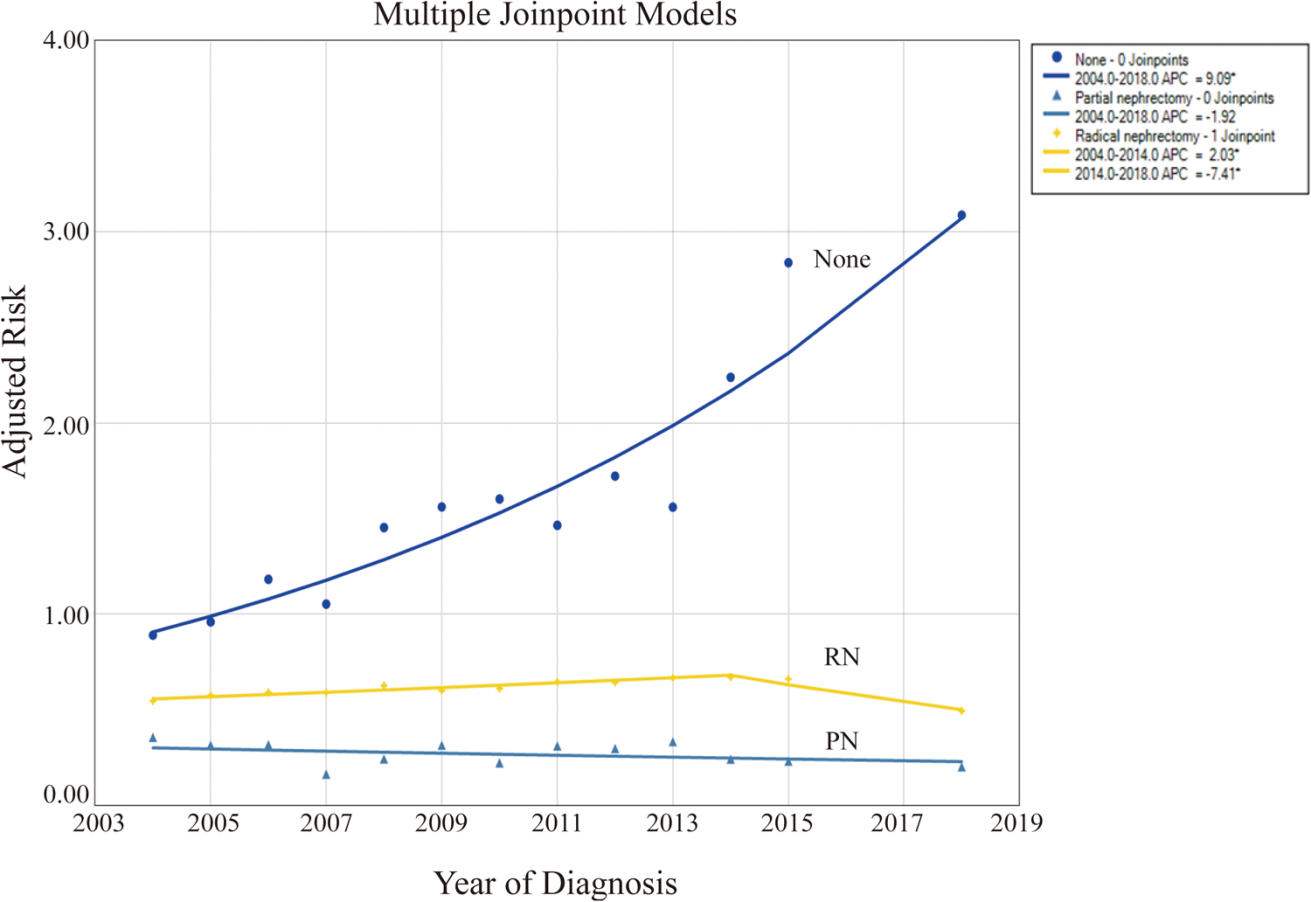
Levels and trends in cancer-specific risk of death (CSRD) undergoing different surgical methods using joinpoint regression analysis. *P < 0.05.

Our findings displayed that patients not undergoing surgery exhibited the highest CSRD levels (0.89–3.09). Patients with PN had the lowest levels (0.17–0.36), whereas those with RN were in between (0.50–0.67). The CSRD was significantly increased in patients not undergoing surgery, with an AAPC of 9.1 (95% CI: 7.1–11.1, *P* < 0.001), which reflects a progressively severe trend over the years (Table 2, Figure 3). By contrast, patients with PN and RN experienced decreasing AAPCs of −1.9 (95% CI: −5.2 to 1.5, *P* = 0.236) and −0.8 (95% CI: −1.4 to −0.1, *P* < 0.001), respectively, indicating an overall trend of gradual improvement. The CSRD among patients with RN indicated the initial increase and the subsequent decline. The APC was 2.0 (95% CI: 1.5–2.6, *P* < 0.001) before 2014 and then shifted to −7.4 (95% CI: −9.6 to −5.2, *P* < 0.001).

The demographic characteristics of patients in the three surgical groups were further examined to explore the potential relationship between surgical methods and CSRD levels. Significant differences were observed among all factors (Table 1). Moreover, 68.5% of the patients not undergoing surgery were ≥65 years old. By contrast, a higher proportion of patients undergoing surgery were <65 years old. Younger individuals have a higher probability of undergoing surgery. Regarding histological grade, the percentages of patients with undetermined grades were 60.1%, 24.6%, and 19.9%, while the values for grade III–IV were 42.4%, 44.2%, and 53.13%, among patients not undergoing surgery, PN, and RN, respectively.

As the surgical complexity increased, patients tended to be diagnosed with more severe grades. In addition, a higher proportion of patients with stage T3 cancer underwent PN. However, the percentage of N stage-positive patients was the lowest (only 1.3%). Patients not undergoing surgery and RN tended to have larger tumors, with median values of 84.0 and 81.0 mm, respectively; these values were significantly larger than those for PN (47.0 mm). In this study, the use frequency of radiotherapy and chemotherapy was not notably high. However, it was prevalent among patients not undergoing surgery or RN. In patients who underwent RN, individuals who received radiotherapy and chemotherapy had a higher CSRD than those who did not receive these therapies.

### 3.3 Stratification analysis of risk factors on CSRD levels and trends

**Figure 4:**
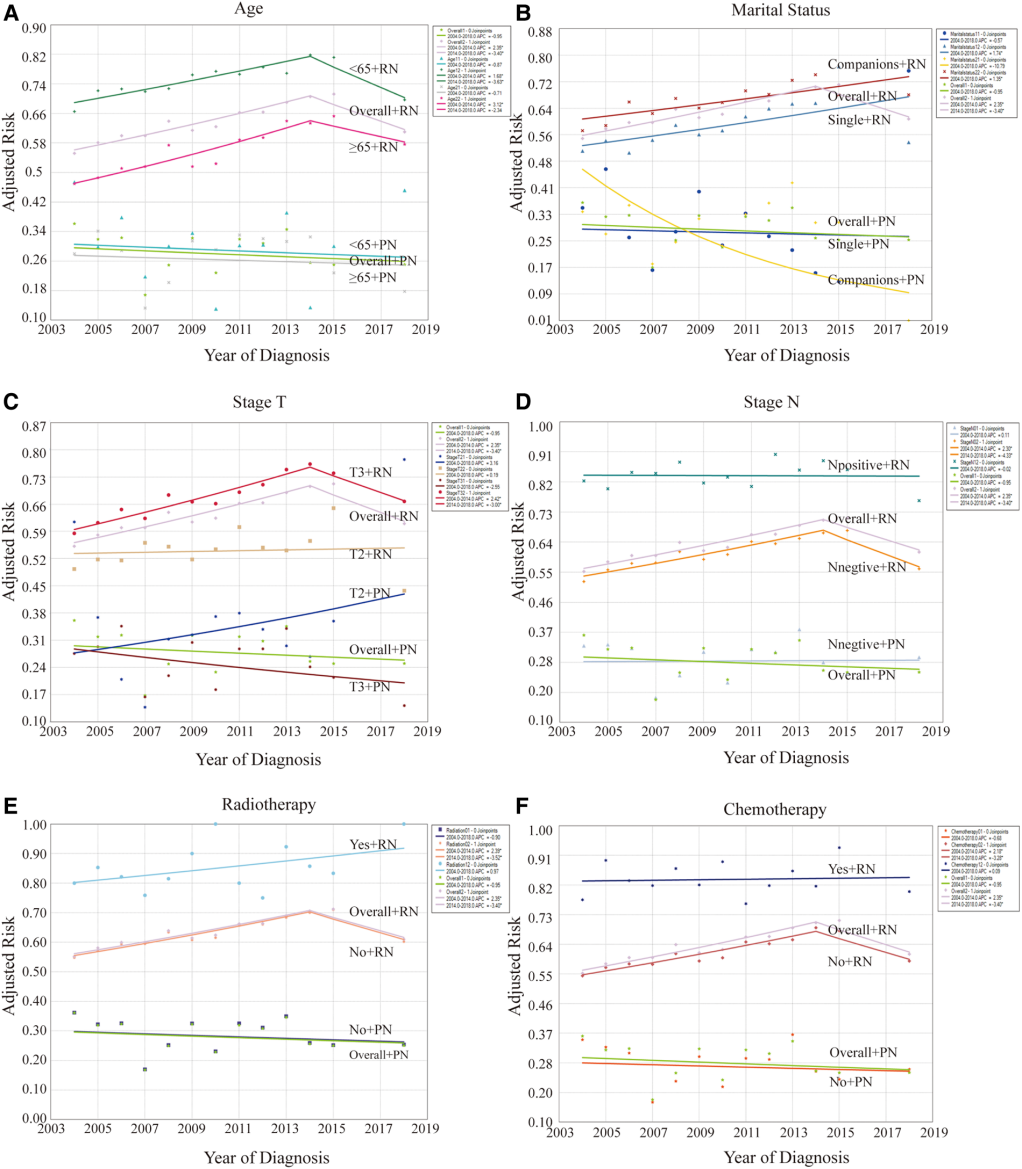
Effect of risk factors on levels and trends in CSRD by stratification analysis between patients with PN and RN. (**A**) Stratified by age. (**B**) Stratified by marital status. (**C**) Stratified by T stages. (**D**) Stratified by N stages. (**E**) Stratified by radiotherapy. (**F**) Stratified by chemotherapy. *P < 0.05.

Logistic regression analysis was first performed to identify the potential risk factors for CSD. Notably, factors such as older age, tumor grade, T and N staging, use of radiotherapy and chemotherapy, and marital status were associated with the risk of CSD among patients. Additionally, using joinpoint regression analysis, the effect of the risk factors on CSRD levels and trends was assessed by different risk factors (Table 3, Figure 4). Across different risk factors and surgical methods, the trends in CSRD generally followed a similar pattern. Patients with RN typically had higher CSRD than those with PN, with no significant differences compared with the overall trends.

**Table 3:** Effect of risk factors on trends in CSRD by stratification analysis.

	Logistic regression		Joinpoint regression			
	exp (coef) [95% CI]	*P*	PN	*P*	RN	*P*
			AAPC		AAPC	
Age	1.53 [1.45, 1.62]*	<0.05				
<65			−0.9 [−7.0, 5.6]	0.80	0.1 [−0.6, 0.9]	0.70
≥65			-0.7 [−5.1, 3.9]	0.70	1.5 [0.1, 3.0]*	<0.05
Marital status	0.92 [0.86, 0.98]*	<0.05				
Single			-0.6 [−7.9, 7.4]	0.90	1.7 [0.3, 3.2]*	<0.05
Married			-19.6 [−25.6, −13.1]*	<0.05	1.4 [0.6, 2.1]*	<0.05
Stage T	1.25 [0.96, 1.63]	0.10				
T2			-0.7 [−13.3, 13.6]	0.90	0.2 [−1.3, 1.7]	0.80
T3			-2.5 [−6.5, 1.5]	0.20	0.8 [−0.0, 1.7]	0.10
*N* positive	4.10 [3.62, 4.65]*	<0.05				
No			0.1 [−3.2, 3.5]	0.90	0.4 [−0.4, 1.1]	0.30
Yes					-0.0 [−0.7, 0.7]	0.90
Beam radiation	4.18 [3.25, 5.40]*	<0.05				
No			-0.9 [−4.1, 2.4]	0.60	0.7 [−0.0, 1.4]	0.10
Yes					1.0 [−0.4, 2.3]	0.10
Chemotherapy	2.51 [2.23, 2.82]*	<0.05				
No			-0.7 [−4.2, 3.0]	0.70	0.6 [−0.1, 1.3]	0.10
Yes					0.1 [−0.8, 1.0]	0.80

PN, partial nephrectomy; RN, radical nephrectomy; AAPC, average annual percentage change.

* *P *< 0.05.

Univariate stratification analysis revealed that the CSRD level was higher in younger patients (<65 years old) across the different surgical groups (Figure 4A). Furthermore, patients with RN had a higher CSRD than those who were single (Figure 4B). When stratified by T staging (Figure 4C), the CSRD level of the RN group was higher than that of the PN group. However, CSRD patterns varied across different staging conditions. Notably, among patients who underwent RN, CSRD was significantly higher for stage T3 cases than for stage T2 cases every year, exhibiting an upward trend. Among patients who underwent PN, the CSRD level of patients with stage T2 was higher than that of those with stage T3 in multiple years. The rate of stage T2 exhibited an increasing trend, whereas that of stage T3 exhibited a decreasing trend. When stratified by N staging (Figure 4D), the result was similar to the whole condition. In patients who underwent RN, the CSRD level was consistently higher in patients who underwent radiotherapy or chemotherapy than in those who did not undergo these therapies (Figures 4E,F). Furthermore, this trend exhibited an upward trend. However, among patients undergoing with PN, this result was not shown due to the relatively small proportion of patients receiving radiotherapy or chemotherapy.

### 3.4 Effect of different surgical methods on overall survival rates and trends

**Figure 5:**
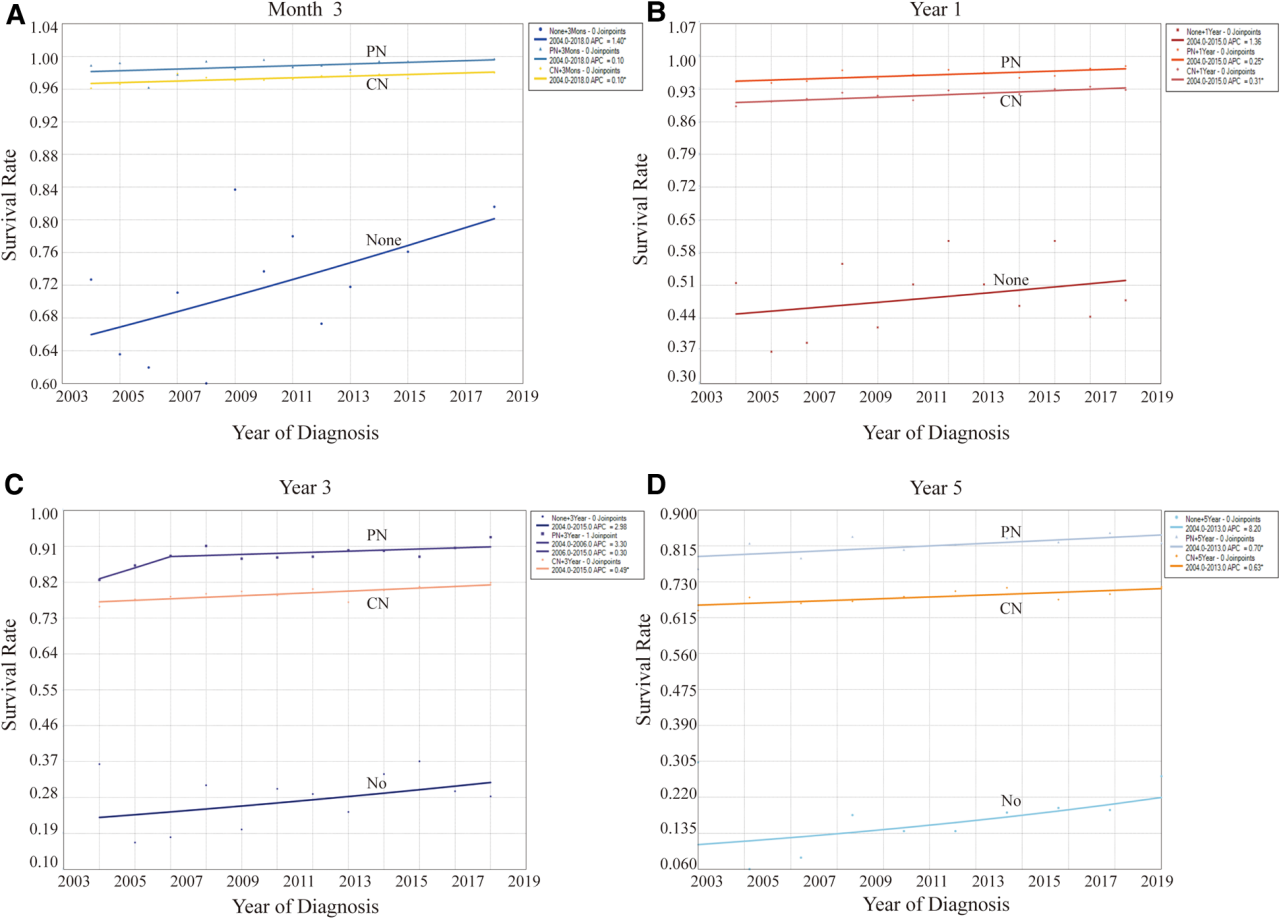
Effect of different surgical methods on levels and trends of the overall survival rates at different follow-up points. (**A**) At month 3. (**B**) At year 1. (**C**) At year 3. (**D**) At year 5. *P < 0.05.

A substantial correlation between cancer survival rates and CSD outcomes was observed, offering a significant advantage in evaluating treatment benefits. This study preliminarily indicated that the survival rates of patients with stage T2-3 KC were significantly high (Table 4, Figure 5). As indicated in Table 4, the 5-year survival rates of individuals not undergoing surgery, RN, and PN were more than 14%, 66%, and 76%, respectively. At different follow-up points, the survival rates and their trends for patients opting for different surgical methods remained largely consistent. In terms of survival rates, patients with PN exhibited the highest rates, followed by those with RN. By contrast, patients not undergoing surgery had the lowest survival rates. This finding underscores the potential beneficial effect of both surgical methods on survival. Regardless of the specific follow-up point, all three surgical methods displayed an upward trend in survival rates. Furthermore, the survival rates of patients with KC, whether treated or not, have displayed improvement over the years, aligning with the trends observed in CSRD.

**Table 4:** Effect of different surgical methods on overall survival rate and trends.

OS	Surgery	2004	2005	2006	2007	2008	2009	2010	2011	2012	2013	2014	2015	2018
3-months	NN	0.727	0.636	0.619	0.711	0.600	0.837	0.737	0.780	0.673	0.718	0.772	0.761	0.816
	PN	0.989	0.992	0.962	0.978	0.994	0.985	0.996	0.987	0.989	0.984	0.994	0.994	0.997
	RN	0.961	0.966	0.969	0.979	0.974	0.970	0.971	0.972	0.976	0.980	0.978	0.973	0.980
1-year	NN	0.515	0.368	0.3871	0.556	0.420	0.512	0.605	0.512	0.466	0.605	0.443	0.478	
	PN	0.946	0.943	0.947	0.970	0.952	0.961	0.971	0.965	0.954	0.958	0.974	0.979	
	RN	0.893	0.903	0.909	0.922	0.916	0.906	0.927	0.912	0.919	0.93	0.935	0.928	
3-year	NN	0.364	0.167	0.181	0.311	0.200	0.302	0.289	0.244	0.339	0.371	0.296	0.283	
	PN	0.826	0.862	0.886	0.911	0.879	0.882	0.884	0.900	0.899	0.884	0.906	0.933	
	RN	0.759	0.777	0.784	0.791	0.797	0.787	0.803	0.770	0.800	0.809	0.809	0.819	
5-year	NN	0.303	0.050	0.0774	0.178	0.140	0.140	0.184	0.195	0.190	0.270			
	PN	0.761	0.821	0.786	0.837	0.806	0.818	0.834	0.824	0.846	0.822			
	RN	0.662	0.693	0.679	0.684	0.695	0.708	0.716	0.688	0.701	0.718			

NN, no nephrectomy; PN, partial nephrectomy; RN, radical nephrectomy.

## 4 Discussion

At present, standardized guidelines and supporting evidence for determining the surgical modality for patients with larger tumors of KC are lacking. Utilizing the SEER database, we observed that RN remains the first surgical choice for patients with stage T2-3 KC. While a positive trend exists for opting for PN, both of them benefited from surgery. Notably, PN exhibited improved survival outcomes and displayed a promising trend for patients in this stage, necessitating further investigation and validation.

RN has been the cornerstone of KC treatment since its introduction in 1969 (18). In the past two decades, researchers have increasingly recognized the benefits of PN over RN for preserving renal function due to the advances in surgical techniques. PN not only maintains comparable cancer-related survival outcomes but may also reduce the risk of mortality from other causes (19). This perspective is bolstered by numerous retrospective studies utilizing data from the SEER database or the National Cancer Database, which affirm the benefits of PN equivalent even over RN for a range of cancer stages (20–26). Furthermore, in addition to garnering substantial popularity, the use of PN has broadened to other indications and is not just limited to solitary kidney, bilateral tumors or to patients at high risk of renal dysfunction (27). Current treatment guidelines recommend PN as the preferred method for patients with stage T1a renal cancer (28). Moreover, for patients with any tumor size, PN should be considered the primary treatment option if technically feasible. However, a phase III prospective randomized controlled trial introduced skepticism regarding the efficacy of PN (29, 30). The analysis of the intention-to-treat population showed that RN offers a slightly better overall survival (OS) than PN for T1-2 stage renal masses. A meta-analysis indicated that this trial was currently the only randomized controlled trial in this field (31). The results of the trial could diminish the popularity of PN, particularly for patients with intractable and large tumors. Nonetheless, some have pointed out that the study mentioned above had certain limitations and the quality of evidence was limited, thereby arguing that the value of PN still merits recognition (32).

PN is recommended predominantly for patients with small renal tumors or those requiring preservation of renal function. The applicability and benefits of PN for large tumors, however, remain debatable (19). Recent meta-analyses indicate that for patients with KC classified as having cT2 stage tumors and above (≥7 cm), PN offers comparable or better cancer-specific outcomes and OS rates relative to RN (8, 33). Nevertheless, PN is associated with an increased risk of complications and adverse side effects, as evidenced by several single-center retrospective analyses (34–36). Although one study had been challenged for population heterogeneity, subsequent adjustments and re-evaluations have reinforced the validity of these findings (37, 38). By contrast, Jeldres and Peycelon have raised concerns for tumors classified as T2 stage and beyond, showing a 5.3-fold increase in the CSM risk associated with PN compared to RN (39, 40). These findings underscored the potential shortcomings of PN and raised ethical questions, with some interpretations suggesting a conflict with the Declaration of Helsinki's principles (41). Subsequent investigations highlighted that the success and efficacy of PN significantly depend on the surgeon's technical expertise rather than purely on tumor grade or stage (42). This highlights a complex dilemma: the choice of PN is related not entirely to the tumor size. In terms of subgroup analysis or survival rate, the results showed a relatively consistent change in levels and trends. The conclusions of this study are fundamentally consistent with previous research, indicating that the surgical choice between PN and RN for T2-3 stage KC indeed requires further prospective exploration (9).

An analysis of the SEER database elucidates the dynamic trends in the utilization of PN vs. RN for KC, demonstrating positive trends but lower levels (20, 21, 24, 25, 43, 44). The results of studies from different institutions and periods vary, but the trends are consistent, with rates ranging from 9.27% to 29% (35, 45, 46). In a study spanning 2000–2018, the application rates of PN demonstrated an average annual percent change of 7.0%, focusing on KC stages T2-3 (46). Although the proportion of PN in this study is lower than what was reported in previous analyses, it aligns with other findings for similar stages. Nonetheless, this trend corroborates with data from the United States between 2005 and 2007, collectively illustrating a positive trajectory in the acceptance and investigation of PN as a viable surgical option for KC stages T2-3 (47).

The selection of surgical intervention for renal cell carcinoma is determined by a multitude of factors. Thus, the acceptance and subsequent application of this recommendation have been progressive (48). This trend underscores the multifaceted influences affecting surgical choices. Several studies based on different databases revealed that the advent of robotic and laparoscopic surgeries has significantly enhanced the application of PN (49–54). These minimally invasive approaches have been pivotal in refining the procedure's efficacy and patient outcomes. Despite this, exploring the target population and expanding its use are necessary. Analysis of data from the American National Cancer Database (55) highlights that the preference for PN diminishes as tumor size escalates. Notably, there exists a 6.1% likelihood of encountering positive surgical margins during PN, correlating with a 31% increase in the risk of all-cause mortality. This concern is particularly pronounced for pT3a tumors, necessitating more rigorous postoperative surveillance for patients undergoing PN with positive margins. Emerging research indicates that the heightened risk of positive surgical margins in PN is less of a consequence of advanced clinical tumor stages and more a result of the burgeoning dependence on minimally invasive surgical methods (44). Despite these insights, a detailed stratified analysis of the surgical techniques in PN remains elusive due to challenges with data access.

In examining factors associated with surgical treatment outcomes, this study utilized logistic regression analysis to identify age and companionship as protective factors against CSRD. These findings align with prior research, which has documented a propensity for younger individuals to prefer PN as a treatment option (21). This investigation reveals that node (N) staging exerts a more pronounced influence on the prognosis of KC than tumor (T) staging. In contrast to these findings, an additional analysis focusing on the determinants of short-term mortality post-surgery identified that both T and N staging significantly impact mortality rates within the initial 30 days of the procedure (56). These disparate conclusions highlight a critical gap in the current understanding and warrant further research to corroborate these observations. The overall trend in CSRD associated with RN in this study indicates a decline. However, an initial increase followed by a subsequent decrease was observed, suggesting that this pattern might be linked to the early extensive application of RN and more severe conditions of patients undergoing this procedure. By contrast, the application of PN has seen an increase, along with a gradual decrease in CSRD rates over time, thereby reinforcing the efficacy of PN in managing KC. The individuals who received radiotherapy and chemotherapy had worse survival, which may indicate that they have a very severe condition that requires radiotherapy and chemotherapy. The decision-making process for surgical intervention in KC encompasses a broad spectrum of considerations, including patient demographics, disease specifics, technological advancements, and economic factors. Considering the inherent selection biases, it becomes imperative to adopt a comprehensive approach in evaluating these surgery types. Consequently, there is a pressing need for prospective studies that can accurately assess the comparative benefits and risks of PN and RN.

In the present study, a retrospective population-based research method was used to identify the relationship and evolving patterns between surgical method choices and prognosis. Undeniably, this approach has both advantages and limitations. One prominent strength of this study is its reliance on substantial real-world data from a large sample population. It facilitates the analysis and illustration of the broader picture and changing patterns regarding the choice of surgical methods and their effect on mortality outcomes in patients with stage T2-3 KC, aligning well with real-world scenarios. However, acknowledging the several limitations of this study is vital. Firstly, the retrospective nature of the study introduces potential bias among different groups. For the surgical choice may be determined by the severe of their condition and other factors as introduced above, which also determined their survival outcomes. Secondly, the study encompasses a broad timeframe, leading to differences in the resolution and recording standards of disease information. Thirdly, the multifaceted nature of the disease introduces complexity to the analysis. Lastly, the absence of specific information on treatment and short-term outcomes as well as a predominant reliance on statistical descriptions in the research methods result in a dearth of confirmatory comparisons, thereby constraining the reliability of the results. The main purpose of this study was to describe the clinical situation and then elucidate its possible effects, rather than efficacy evaluation. Therefore, population bias has a little impact on this study, which may not conflict with the main purpose.

In conclusion, treating stage T2-3 KC with PN is garnering increasing attention, presenting significant clinical relevance. While tumor size plays a critical role in guiding the choice of kidney-preserving surgical approaches, it should be considered a contributing factor rather than an absolute determinant. Emphasizing and validating the role of PN in stage T2-3 KC is vital in the future. This study provides valuable insights for precisely selecting surgical approaches and future research designs in this patient population.

## Data Availability

Publicly available datasets were analyzed in this study. This data can be found here: SEER.

## References

[1] Sung H Ferlay J Siegel RL Laversanne M Soerjomataram I Jemal A Global cancer statistics 2020: GLOBOCAN estimates of incidence and mortality worldwide for 36 cancers in 185 countries. CA Cancer J Clin. (2021) 71(3):209–49. 10.3322/caac.21660 33538338

[2] Bukavina L Bensalah K Bray F Carlo M Challacombe B Karam JA Epidemiology of renal cell carcinoma: 2022 update. Eur Urol. (2022) 82(5):529–42. 10.1016/j.eururo.2022.08.019 36100483

[3] Wasserman M Sobel D Pareek G. Choice of surgical options in kidney cancer and surgical complications. Semin Nephrol. (2020) 40(1):42–8. 10.1016/j.semnephrol.2019.12.005 32130965

[4] Han JS Huang WC. Impact of kidney cancer surgery on oncologic and kidney functional outcomes. Am J Kidney Dis. (2011) 58(5):846–54. 10.1053/j.ajkd.2011.07.021 21956016

[5] Russo P. Oncological and renal medical importance of kidney-sparing surgery. Nat Rev Urol. (2013) 10(5):292–9. 10.1038/nrurol.2013.34 23459033

[6] Small AC Tsao CK Moshier EL Gartrell BA Wisnivesky JP Godbold J Trends and variations in utilization of nephron-sparing procedures for stage I kidney cancer in the United States. World J Urol. (2013) 31(5):1211–7. 10.1007/s00345-012-0873-6 22622394 PMC4744479

[7] Campbell SC Clark PE Chang SS Karam JA Souter L Uzzo RG. Renal mass and localized renal cancer: evaluation, management, and follow-up: AUA guideline: part I. J Urol. (2021) 206(2):199–208. 10.1097/JU.0000000000001911 34115547

[8] Li J Zhang Y Teng Z Han Z. Partial nephrectomy versus radical nephrectomy for CT2 or greater renal tumors: a systematic review and meta-analysis. Minerva Urol Nefrol. (2019) 71(5):435–44. 10.23736/S0393-2249.19.03470-2 31287256

[9] Mir MC Derweesh I Porpiglia F Zargar H Mottrie A Autorino R. Partial nephrectomy versus radical nephrectomy for clinical T1b and T2 renal tumors: a systematic review and meta-analysis of comparative studies. Eur Urol. (2017) 71(4):606–17. 10.1016/j.eururo.2016.08.060 27614693

[10] Kopp RP Mehrazin R Palazzi KL Liss MA Jabaji R Mirheydar HS Survival outcomes after radical and partial nephrectomy for clinical T2 renal tumours categorised by R.E.N.A.L. nephrometry score. BJU Int. (2014) 114(5):708–18. 10.1111/bju.12580 24274650

[11] Kutikov A Uzzo RG. The R.E.N.A.L. nephrometry score: a comprehensive standardized system for quantitating renal tumor size, location and depth. J Urol. (2009) 182(3):844–53. 10.1016/j.juro.2009.05.035 19616235

[12] Mir MC Pavan N Capitanio U Antonelli A Derweesh I Rodriguez-Faba O Partial versus radical nephrectomy in very elderly patients: a propensity score analysis of surgical, functional and oncologic outcomes (RESURGE project). World J Urol. (2020) 38(1):151–8. 10.1007/s00345-019-02665-2 30937569

[13] Marchioni M Preisser F Bandini M Nazzani S Tian Z Kapoor A Comparison of partial versus radical nephrectomy effect on other-cause mortality, cancer-specific mortality, and 30-day mortality in patients older than 75 years. Eur Urol Focus. (2019) 5(3):467–73. 10.1016/j.euf.2018.01.007 29398456

[14] Miller C Raza SJ Davaro F May A Siddiqui S Hamilton ZA. Trends in the treatment of clinical T1 renal cell carcinoma for octogenarians: analysis of the national cancer database. J Geriatr Oncol. (2019) 10(2):285–91. 10.1016/j.jgo.2018.11.010 30528544

[15] McIntosh AG Parker DC Egleston BL Uzzo RG Haseebuddin M Joshi SS Prediction of significant estimated glomerular filtration rate decline after renal unit removal to aid in the clinical choice between radical and partial nephrectomy in patients with a renal mass and normal renal function: nomogram to predict post nephrectomy EGFR. BJU Int. (2019) 124(6):999–1005. 10.1111/bju.14839 31145523 PMC7654970

[16] Beyer K Barod R Fox L Van Hemelrijck M Kinsella N. The current evidence for factors that influence treatment decision making in localized kidney cancer: a mixed methods systematic review. J Urol. (2021) 206(4):827–39. 10.1097/JU.0000000000001901 34111958

[17] Tsivian M Joyce DD Packiam VT Lohse CM Boorjian SA Potretzke TA Unplanned conversion from partial to radical nephrectomy: an analysis of incidence, etiology, and risk factors. J Urol. (2022) 208(5):960–8. 10.1097/JU.0000000000002837 35748729

[18] Robson CJ Churchill BM Anderson W. The results of radical nephrectomy for renal cell carcinoma. 1969. J Urol. (2002) 167(2 Pt 2):873–7. 10.1016/S0022-5347(02)80286-5 11905914

[19] Nahar B Gonzalgo ML. What is the current role of partial nephrectomy for T2 tumors? Can J Urol. (2017) 24(2):8698–704. PMID: . 28436354

[20] Shum CF Bahler CD Sundaram CP. Matched comparison between partial nephrectomy and radical nephrectomy for T2 N0 M0 tumors, a study based on the national cancer database. J Endourol. (2017) 31(8):800–5. 10.1089/end.2017.0190 28486848

[21] Zini L Perrotte P Capitanio U Jeldres C Shariat SF Antebi E Radical versus partial nephrectomy: effect on overall and noncancer mortality. Cancer. (2009) 115(7):1465–71. 10.1002/cncr.24035 19195042

[22] Tan HJ Norton EC Ye Z Hafez KS Gore JL Miller DC. Long-term survival following partial vs radical nephrectomy among older patients with early-stage kidney cancer. JAMA. (2012) 307(15):1629–35. 10.1001/jama.2012.475 22511691 PMC3864575

[23] Meskawi M Becker A Bianchi M Trinh QD Roghmann F Tian Z Partial and radical nephrectomy provide comparable long-term cancer control for T1b renal cell carcinoma. Int J Urol. (2014) 21(2):122–8. 10.1111/iju.12204 23819700

[24] Badalato GM Kates M Wisnivesky JP Choudhury AR McKiernan JM. Survival after partial and radical nephrectomy for the treatment of stage T1bN0M0 renal cell carcinoma (RCC) in the USA: a propensity scoring approach. BJU Int. (2012) 109(10):1457–62. 10.1111/j.1464-410X.2011.10597.x 21933334

[25] Alanee S Nutt M Moore A Holland B Dynda D Wilber A Partial nephrectomy for T2 renal masses: contemporary trends and oncologic efficacy. Int Urol Nephrol. (2015) 47(6):945–50. 10.1007/s11255-015-0975-3 25864101

[26] Pecoraro A Amparore D Manfredi M Piramide F Checcucci E Tian Z Partial vs. radical nephrectomy in non-metastatic pT3a kidney cancer patients: a population-based study. Minerva Urol Nephrol. (2022) 74(4):445–51. 10.23736/S2724-6051.22.04680-8 35147387

[27] Mazzone E Nazzani S Preisser F Tian Z Marchioni M Bandini M Partial nephrectomy seems to confer a survival benefit relative to radical nephrectomy in metastatic renal cell carcinoma. Cancer Epidemiol. (2018) 56:118–25. 10.1016/j.canep.2018.08.006 30173050

[28] Ljungberg B Bensalah K Canfield S Dabestani S Hofmann F Hora M EAU guidelines on renal cell carcinoma: 2014 update. Eur Urol. (2015) 67(5):913–24. 10.1016/j.eururo.2015.01.005 25616710

[29] Van Poppel H Da Pozzo L Albrecht W Matveev V Bono A Borkowski A A prospective, randomised EORTC intergroup phase 3 study comparing the oncologic outcome of elective nephron-sparing surgery and radical nephrectomy for low-stage renal cell carcinoma. Eur Urol. (2011) 59(4):543–52. 10.1016/j.eururo.2010.12.013 21186077

[30] Scosyrev E Messing EM Sylvester R Campbell S Van Poppel H. Renal function after nephron-sparing surgery versus radical nephrectomy: results from EORTC randomized trial 30904. Eur. Urol. (2014) 65(2):372–7. 10.1016/j.eururo.2013.06.044 23850254

[31] Kunath F Schmidt S Krabbe LM Miernik A Dahm P Cleves A Partial nephrectomy versus radical nephrectomy for clinical localised renal masses. Cochrane Database Syst Rev. (2017) 5(5):CD012045. 10.1002/14651858.CD012045.pub2 28485814 PMC6481491

[32] Sun M Hansen J Karakiewicz PI. Re: Hendrik Van Poppel, Luigi Da Pozzo, Walter Albrecht, et al. A prospective, randomized EORTC intergroup phase 3 study comparing the oncologic outcome of elective nephron-sparing surgery and radical nephrectomy for low-stage renal cell carcinoma. Eur Urol. (2012) 61(4):e37–8. 10.1016/j.eururo.2011.11.048 22172372

[33] Deng W Chen L Wang Y Liu X Wang G Fu B. Partial nephrectomy versus radical nephrectomy for large (≥7 cm) renal tumors: a systematic review and meta-analysis. Urol Oncol. (2019) 37(4):263–72. 10.1016/j.urolonc.2018.12.015 30704957

[34] Breau RH Crispen PL Jimenez RE Lohse CM Blute ML Leibovich BC. Outcome of stage T2 or greater renal cell cancer treated with partial nephrectomy. J Urol. (2010) 183(3):903–8. 10.1016/j.juro.2009.11.037 20083271

[35] Klett DE Tsivian M Packiam VT Lohse CM Ahmed ME Potretzke TA Partial versus radical nephrectomy in clinical T2 renal masses. Int J Urol. (2021) 28(11):1149–54. 10.1111/iju.14664 34382267

[36] Margulis V Tamboli P Jacobsohn KM Swanson DA Wood CG. Oncological efficacy and safety of nephron-sparing surgery for selected patients with locally advanced renal cell carcinoma. BJU Int. (2007) 100(6):1235–9. 10.1111/j.1464-410X.2007.07225.x 17979923

[37] Cimen HI Canda AE Balbay MD. Re: outcome of stage T2 or greater renal cell cancer treated with partial nephrectomy: R. H. Breau, P. L. Crispen, R. E. Jimenez, C. M. Lohse, M. L. Blute and B. C. Leibovich J Urol 2010; 183: 903–908. J Urol. (2010) 184(5):2212–3. 10.1016/j.juro.2010.06.123 20083271

[38] Okhawere KE Grauer R Zuluaga L Meilika KN Ucpinar B Beksac AT Operative and oncological outcomes of salvage robotic radical and partial nephrectomy: a multicenter experience. J Robot Surg. (2023) 17(4):1579–85. 10.1007/s11701-023-01538-6 36928751

[39] Jeldres C Patard JJ Capitanio U Perrotte P Suardi N Crepel M Partial versus radical nephrectomy in patients with adverse clinical or pathologic characteristics. Urology. (2009) 73(6):1300–5. 10.1016/j.urology.2008.08.492 19376568

[40] Peycelon M Hupertan V Comperat E Renard-Penna R Vaessen C Conort P Long-term outcomes after nephron sparing surgery for renal cell carcinoma larger than 4 cm. J Urol. (2009) 181(1):35–41. 10.1016/j.juro.2008.09.025 19012929

[41] Campi R Bertolo R Minervini A, European Association of Urology Young Academic Urologists Renal Cancer Working Group. Reply to Takeshi Takahashi’s letter to the editor re: Riccardo Campi, Riccardo Bertolo, Andrea Minervini, European association of urology young academic urologists renal cancer working group. Re: partial versus radical nephrectomy in clinical T2 renal masses. Klett DE, Tsivian M, Packiam VT, et al. Int J urol. 2021;28:1149–54. Eur Urol 2021;80;760–2. Partial nephrectomy for T2 kidney cancer might violate the declaration of Helsinki: re-envisioning the value of clinical research to foster the progress of evidence-based urology: the case of partial nephrectomy for cT2 renal masses. Eur Urol. (2022) 81(2):e46–7. 10.1016/j.eururo.2021.11.021 34887115

[42] Hansen J Sun M Bianchi M Rink M Tian Z Hanna N Assessment of cancer control outcomes in patients with high-risk renal cell carcinoma treated with partial nephrectomy. Urology. (2012) 80(2):347–53. 10.1016/j.urology.2012.04.043 22698478

[43] Dulabon LM Lowrance WT Russo P Huang WC. Trends in renal tumor surgery delivery within the United States. Cancer. (2010) 116(10):2316–21. 10.1002/cncr.24965 20225227 PMC4235157

[44] Fero K Hamilton ZA Bindayi A Murphy JD Derweesh IH. Utilization and quality outcomes of cT1a, cT1b and cT2a partial nephrectomy: analysis of the national cancer database. BJU Int. (2018) 121(4):565–74. 10.1111/bju.14055 29032581

[45] Patel SG Penson DF Pabla B Clark PE Cookson MS Chang SS National trends in the use of partial nephrectomy: a rising tide that has not lifted all boats. J Urol. (2012) 187(3):816–21. 10.1016/j.juro.2011.10.173 22248514

[46] Simone G De Nunzio C Ferriero M Cindolo L Brookman-May S Papalia R Trends in the use of partial nephrectomy for CT1 renal tumors: analysis of a 10-yr European multicenter dataset. Eur J Surg Oncol EJSO. (2016) 42(11):1729–35. 10.1016/j.ejso.2016.03.022 27106494

[47] Kowalczyk KJ Choueiri TK Hevelone ND Trinh QD Lipsitz SR Nguyen PL Comparative effectiveness, costs and trends in treatment of small renal masses from 2005 to 2007. BJU Int. (2013) 112(4):e273–80. 10.1111/j.1464-410X.2012.11776.x 23452093

[48] Bjurlin MA Walter D Taksler GB Huang WC Wysock JS Sivarajan G National trends in the utilization of partial nephrectomy before and after the establishment of AUA guidelines for the management of renal masses. Urology. (2013) 82(6):1283–9. 10.1016/j.urology.2013.07.068 24295245 PMC3852430

[49] Okhawere KE Milky G Razdan S Shih IF Li Y Zuluaga L One-year healthcare costs after robotic-assisted and laparoscopic partial and radical nephrectomy: a cohort study. BMC Health Serv Res. (2023) 23(1):1099. 10.1186/s12913-023-10111-8 37838666 PMC10576279

[50] Okhawere KE Pandav K Grauer R Wilson MP Saini I Korn TG Trends in the surgical management of kidney cancer by tumor stage, treatment modality, facility type, and location. J Robot Surg. (2023) 17(5):2451–60. 10.1007/s11701-023-01664-1 37470910

[51] Suek T Davaro F Raza SJ Hamilton Z. Robotic surgery for cT2 kidney cancer: analysis of the national cancer database. J Robot Surg. (2022) 16(3):723–9. 10.1007/s11701-021-01300-w 34435278

[52] Ingels A Bensalah K Beauval JB Paparel P Rouprêt M Lang H Comparison of open and robotic-assisted partial nephrectomy approaches using multicentric data (UroCCR-47 study). Sci Rep. (2022) 12(1):18981. 10.1038/s41598-022-22912-8 36347900 PMC9643517

[53] Poon SA Silberstein JL Chen LY Ehdaie B Kim PH Russo P. Trends in partial and radical nephrectomy: an analysis of case logs from certifying urologists. J Urol. (2013) 190(2):464–9. 10.1016/j.juro.2013.02.094 23454156 PMC3710515

[54] Liu W Zhang E Zhang M. Current application of navigation systems in robotic-assisted and laparoscopic partial nephrectomy: focus on the improvement of surgical performance and outcomes. Ann Surg Oncol. (2024) 31(3):2163–72. 10.1245/s10434-023-14716-5 38063985

[55] Ryan ST Patel DN Ghali F Patel SH Sarkar R Yim K Impact of positive surgical margins on survival after partial nephrectomy in localized kidney cancer: analysis of the national cancer database. Minerva Urol Nephrol. (2021) 73(2):233–44. 10.23736/S2724-6051.20.03728-5 32748614

[56] Fontenil A Bigot P Bernhard JC Beauval JB Soulié M Charles T Who is dying after nephrectomy for cancer? Study of risk factors and causes of death after analyzing morbidity and mortality reviews (UroCCR-33 study). Prog En Urol. (2019) 29(5):282–7. 10.1016/j.purol.2019.02.009 30962141

